# Zanubrutinib-induced petechial ecchymotic reaction in a previously irradiated area in a patient with chronic lymphocytic leukemia

**DOI:** 10.1016/j.jdcr.2024.01.020

**Published:** 2024-02-24

**Authors:** Serena M. Vilasi, Lydia A. Luu, Mary M.B. Noland

**Affiliations:** a School of Medicine, University of Virginia, Charlottesville, Virginia; b Department of Dermatology, University of Virginia Health System, Charlottesville, Virginia

**Keywords:** Bruton’s tyrosine kinase inhibitor, chronic lymphocytic leukemia, locus minoris resistenciae, petechial ecchymotic reaction, zanubrutinib, zanubrutinib-induced rash, zanubrutinib-induced skin reaction, zanubrutinib radiation dermatitis, zanubrutinib skin

## Introduction

The first Bruton’s tyrosine kinase (BTK) inhibitor ibrutinib was approved by the US Food and Drug Administration (FDA) in 2014 for treatment of chronic lymphocytic leukemia (CLL). First-generation BTK inhibitors have revolutionized the treatment of lymphoproliferative disorders but carry risk of dermatologic toxicities such as ecchymoses, rash, and skin infection.^1^

Zanubrutinib is a second-generation BTK inhibitor developed to have a greater specificity and fewer off-target effects.^1^ The FDA approved zanubrutinib in January 2023 for the treatment of CLL after studies showed that it achieved superior overall response rates in patients with relapsed or refractory CLL or small lymphocytic lymphoma compared with ibrutinib.^2^

Dermatologic toxicities in patients receiving zanubrutinib have not been thoroughly characterized to date. Here, we present a case of a petechial ecchymotic reaction to zanubrutinib localized to a previously irradiated area.

## Case report

A 69-year-old female patient presented to a dermatology clinic with discoloration of the right breast for 2 months. The patient denied pain, pruritus, or other constitutional symptoms. She had a history of high-grade invasive carcinoma, with mixed ductal and lobular features, in the right breast 3 years ago, which was treated with lumpectomy and radiation therapy in 2020, adjuvant chemotherapy with docetaxel, carboplatin, trastuzumab, and pertuzumab in 2021, and is currently receiving letrozole since 2022. She also has a history of CLL diagnosed 11 years ago and was initially managed receiving ibrutinib, but due to recurrence of CLL, she began receiving zanubrutinib 160 mg twice daily 4 months ago.

**Fig 1:**
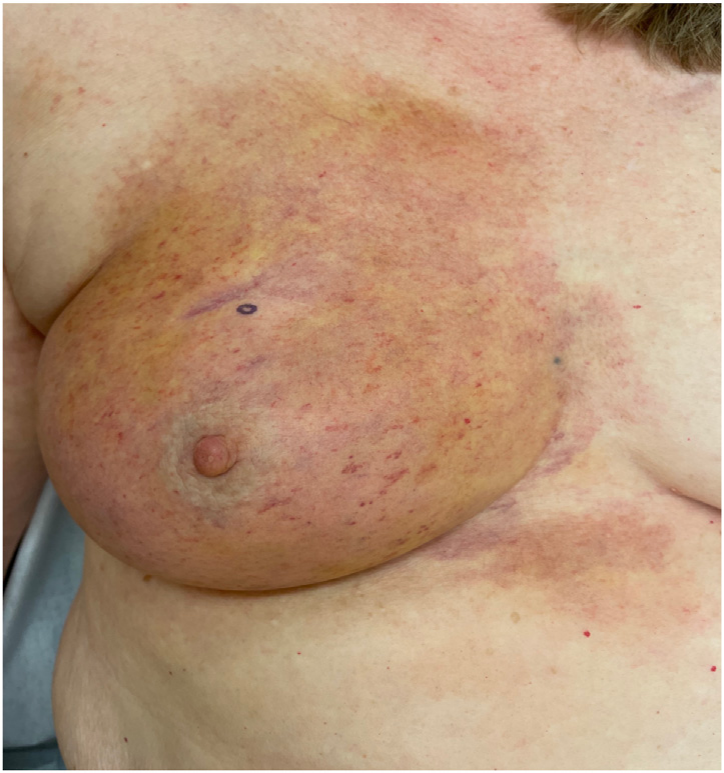
Zanubrutinib-induced petechial ecchymotic reaction. Physical examination revealed yellow-brown macular discoloration over the right breast extending onto the intermammary skin and upper portion of the abdomen with petechiae scattered throughout, along with overlying atrophy, telangiectasias, and hyper- and hypopigmentation. No induration, peau d’orange change, or nodules were appreciated.

On examination, there was well-demarcated, yellow-brown macular discoloration over the right breast that extended onto the intermammary skin and upper portion of the abdomen with petechiae scattered throughout. The skin showed changes of radiation dermatitis with some atrophy, telangiectasias, and both hyper- and hypopigmentation. Induration, peau d’orange change, or nodules were not present (Fig 1). There was a scar over the right breast from prior lumpectomy, and the right breast appeared smaller than the left. There were no skin changes noted elsewhere, and the patient’s platelet count was within normal limits (197 U/μL). The differential diagnosis included hemosiderin deposition in an area of radiation dermatitis, cutaneous breast cancer, and angiosarcoma.

A punch biopsy was obtained from the right breast. Histopathologic examination revealed mild perivascular inflammation and focal erythrocyte extravasation in the papillary dermis with minimal focal iron deposition. The blood vessels showed no architectural complexity or atypia, and there was no increased dermal mucin. Immunohistochemical staining for C-MYC was negative. Overall, the biopsy was consistent with hemosiderin deposition or ecchymosis in the area of radiation dermatitis exacerbated by zanubrutinib therapy. She was reassured of these benign skin changes and continued receiving zanubrutinib with planned follow-up in dermatology clinic in the near future.

## Discussion

Adverse skin reactions are among the most common toxicities attributed to BTK inhibitors.^1^ Second-generation BTK inhibitors such as zanubrutinib have a more selective binding profile, minimizing off-target inhibition and toxicity.^1^ The dermatologic toxicities and management strategies associated with zanubrutinib have not yet been fully characterized.^1^

The most common dermatologic toxicities of BTK inhibitors are bruising or skin ecchymoses, which occur in 23% to 33% of patients treated with ibrutinib and 43% of patients treated with zanubrutinib, likely due to inhibition of BTK and Tec kinases, both of which are involved in platelet activation and aggregation.^1^ These skin changes are commonly located on areas of microtrauma, UV exposure, or skin atrophy and do not require treatment disruption. Treatment involves emollients and avoiding cutaneous microtrauma. Importantly, occurrence of bruising or ecchymosis does not indicate higher risk of more severe bleeding events that are associated with BTK-inhibitor therapy.^3^

In addition to bruising, rash was reported in 13% to 27% of patients treated with ibrutinib and 13% to 18% of patients treated with zanubrutinib.^1^ Nocco et al^3^ found 5 rash morphologies associated with ibrutinib: (1) nonpalpable petechial rash, (2) leukocytoclastic vasculitis–like pruritic palpable purpura, (3) pityriasis rosea–like rash, (4) papulopustular rash, and (5) painless nonpruritic edematous papules. The petechial rash is likely due to BTK-mediated platelet dysfunction and often presents months after treatment initiation.^3^ Edematous or purpuric papular rashes likely represent an immune-mediated drug reaction, begin in the first weeks of drug initiation, and are more common in patients with a history of drug hypersensitivity.^3^ The pityriasis rosea–like rash presents most often on the trunk as violaceous, scaly, pruritic plaques, or papules, is likely due to off-target inhibition of c-Kit and platelet-derived growth factor receptor,^3^ and can be managed with corticosteroids.^1^ Singer et al^4^ demonstrated that the papulopustular rash is likely due to off-target inhibition of epidermal growth factor receptor, presents in the first few weeks of treatment, and commonly has associated photosensitivity. Management requires ruling out *Staphylococcus aureus* infection and initiating treatment with tetracyclines and topical corticosteroids.^1^

Petechial or ecchymotic skin changes attributable to BTK inhibitors usually resolve over weeks.^1^ One study demonstrated rash resolution after a median of 21 days with topical corticosteroids, oral antihistamines, and no BTK-inhibitor cessation, whereas another report demonstrated resolution in 14 days with oral steroids, antihistamines, and temporary BTK-inhibitor cessation.^5^^,^^6^ For more persistent or severe rash, clinicians may consider temporary suspension of BTK-inhibitor therapy and treating with topical or oral corticosteroids until cutaneous symptoms resolve.^3^^,^^5^^,^^7^

As this patient’s skin changes occurred in an area that was previously irradiated, it may be a manifestation of locus minoris resistenciae (lmr). Lmr refers to a region of the body at increased risk of inflammatory processes due to prior trauma.^8^ The pathophysiology of lmr is unclear, but may be a result of structural abnormalities in the skin after injury.^9^ Previous irradiation of the breast may have left the area more susceptible to toxic effects of BTK-inhibitor therapy, resulting in a well-demarcated area of skin change.

In this paper, we describe a petechial ecchymotic reaction in a previously irradiated area as a reaction of zanubrutinib, a recently FDA-approved treatment for CLL. Although this reaction is benign and does not require treatment discontinuation, dermatologic toxicity is a major cause of treatment disruption in patients taking BTK inhibitors and can lead to resistance to therapy.^3^^,^^7^ Thus, identification and management of cutaneous toxicities are vital in preventing treatment disruption or discontinuation and poor clinical outcomes in this patient population.

## Conflicts of interest

None disclosed.
